# Relief of pain associated with spasticity in adult patients after treatment with onabotulinumtoxinA: Post hoc observational results from the ASPIRE study

**DOI:** 10.1002/pmrj.70013

**Published:** 2025-09-30

**Authors:** Jörg Wissel, Cassandra List, Marc Schwartz, Mariana Nelson, Tiziana Musacchio, Esther Duarte

**Affiliations:** ^1^ Neurology and Psychosomatic at Wittenbergplatz Berlin Germany; ^2^ Brooks Rehabilitation Jacksonville Florida USA; ^3^ MS Biostatistics, LLC Clermont Florida USA; ^4^ AbbVie Irvine California USA; ^5^ AbbVie, Inc. Rome Italy; ^6^ Hospital del Mar Barcelona Spain

## Abstract

**Background:**

Pain is often observed in patients with spasticity, but little is known about the relationship between pain and spasticity and the effectiveness of treating pain with botulinum toxins in these patients.

**Objective:**

To explore onabotulinumtoxinA (onabotA) use and pain relief in patients with spasticity with pain at baseline.

**Design:**

Subanalysis of a 2‐year multicenter, prospective, observational study (ASPIRE [Adult Spasticity International Registry], NCT01930786).

**Setting:**

Fifty‐four international clinical sites.

**Participants:**

Adults with spasticity (*N* = 494) and pain at baseline (Numeric Pain Rating Scale [NPRS]>0) across multiple etiologies and age groups.

**Intervention:**

OnabotA administered at clinician's discretion.

**Main Outcomes:**

OnabotA use, pain measured with the NPRS and Disability Assessment Scale (DAS), patient‐ and physician‐reported satisfaction, and safety.

**Results:**

Of 730 patients who received ≥1 onabotA dose in ASPIRE, 494 (68%) had baseline pain (mean age, 54 years; stroke, 56%; naïve to onabotA, 38%; NPRS ≥5, 65%). Average onabotA dose per treatment session (Tx) ranged from 345 to 463 U. Pain reduction from baseline was observed across all Tx; mean NPRS decreased from 5.4 to 2.6 at Tx8, model estimated mean NPRS was significantly reduced after each Tx (Tx1–7, *p* ≤ .001; Tx8, *p* ≤ .005), and a high proportion achieved clinically meaningful pain reductions across Tx1–4 (mean NPRS decreased by ≥30% for 49%–59%, by ≥50% for 40%–49%, and by ≥70% for 24%–34% of patients). Relief of pain was supported by significant improvements from baseline on the DAS pain subscale across most Tx with most patients/physicians being satisfied with onabotA treatment. Overall, 15 patients (3%) reported 17 treatment‐related adverse events (TRAEs), and 2 patients (0.4%) reported 3 serious TRAEs.

**Conclusion:**

In patients with spasticity experiencing pain, long‐term onabotA treatment demonstrated consistent clinically meaningful reductions in pain, reduced pain disability on DAS, and high patient and physician satisfaction with no new safety signals identified, regardless of prior onabotA treatment or age groups.

## INTRODUCTION

Spasticity is a central nervous system condition characterized by disordered sensorimotor control, resulting from damage to the upper motor neurons.^1^, ^2^ It manifests as intermittent or sustained involuntary activation of muscles, leading to abnormal postures and limb pain.^3^, ^4^ The underlying etiologies of spasticity are diverse, including ischemic or hemorrhagic stroke, traumatic brain injury, spinal cord injury, multiple sclerosis, cerebral palsy, and various other neurological disorders.^5^, ^6^, ^7^ One of the significant challenges faced by patients with spasticity is the presence of pain, which affects their health‐related quality of life, increases the burden placed on their caregivers, and imposes substantial societal and health care costs.^8^, ^9^, ^10^, ^11^, ^12^, ^13^, ^14^ Previous studies have reported a high prevalence of pain in patients with spasticity, indicating that up to 65% of patients experience pain related to their spasticity.^11^, ^15^, ^16^ Post stroke, patients reported prevalence of pain between 10% and 70%.^11^, ^17^ Pain in patients with spasticity can be categorized into nociceptive pain, which includes musculoskeletal pain, and^18^ neuropathic pain,^19^ which results from nerve damage and is characterized by central neuropathic pain^20^ originating in the brain or spinal cord (eg, following a stroke or multiple sclerosis)^21^ and peripheral neuropathic pain originating from peripheral nerves, such as in spinal cord injury.^22^ Mixed pain, a combination of nociceptive and neuropathic pain components, also commonly occurs.^23^

OnabotulinumtoxinA (OnabotA) emerged as a recommended treatment of choice in patients with spasticity due to its well‐established safety, long‐term tolerability, efficacy across a range of doses, improvement in symptoms and quality of life measures, high patient and physician satisfaction with the treatment, and the potential to reduce costs and health care use.^24^, ^25^, ^26^, ^27^ OnabotA is approved for the treatment of upper and lower limb spasticity in both pediatric and adult patients.^28^ Although the mechanism of action of onabotA in reducing pain in patients with spasticity is not fully understood, it is known to regulate neurotransmitter release at the neuromuscular junction and decrease muscle activity,^29^ thereby improving associated symptoms (eg, pain)^30^ and impaired function and goal attainment.^29^, ^31^ OnabotA has demonstrated efficacy in reducing pain in other therapeutic indications not associated with spasticity such as prophylaxis of headaches in adults with chronic migraine (CM)^32^, ^33^ and in patients with cervical dystonia (CD),^34^, ^35^, ^36^ through multiple mechanisms.^37^, ^38^ In CM, onabotA is thought to alleviate pain by inhibiting the release of pain mediators, such as substance P, calcitonin gene‐related peptide, and glutamate, from peripheral nerve terminals.^33^

Despite the high prevalence of pain in patients with spasticity, only a few real‐world observational studies have explored the potential of onabotA treatment in patients with spasticity to provide pain relief as an additional benefit to spasticity‐associated muscle relaxation.^7^, ^11^, ^12^, ^31^, ^39^, ^40^ A better understanding of the effectiveness of onabotA on pain management in patients with spasticity is needed to inform treatment decisions in clinical practice.^11^, ^41^, ^42^ This subgroup analysis of the large, observational, Adult Spasticity International Registry (ASPIRE) study aims to fill this gap by evaluating onabotA use patterns and effectiveness in patients with spasticity with pain (Numeric Pain Rating Scale [NPRS]>0) at baseline, more specifically assessing the effect of long‐term onabotA treatment on pain, as measured by NPRS, pain disability subscale measured by the Disability Assessment Scale (DAS), and patient‐ and physician‐reported satisfaction outcomes. Patients with spasticity included in this study were of diverse etiologies, naïve and nonnaïve to prior onabotA treatment, and comprised younger and older age groups, which is reflective of real‐world practices. This analysis will help address the potential role of onabotA in pain reduction in patients with spasticity and increase awareness among physicians to concurrently address pain management in their spasticity treatment programs.

## METHODS

### Study design and setting

This post hoc analysis on pain, assessed by patient‐reported NPRS and physician‐reported DAS on pain area of functional disability, as well as patient‐ and physician‐reported satisfaction in patients with spasticity and pain at baseline (NPRS>0) (Supplemental Figure S1), uses data from a large international (Asia, Europe, and North America), multicenter (54 clinical sites), prospective, observational registry (ASPIRE, NCT01930786) that reported outcomes on lower limb (LL), upper limb (UL),^5^, ^41^, ^42^, ^43^ and hemiparetic patients.^31^ The ASPIRE study data collection extended over 108 weeks, encompassing a 96‐week study period and an additional 12‐week follow‐up period. Study design and setting were first described in Franscisco et al., 2017.^5^, ^41^, ^42^, ^43^, ^44^, ^45^ Before participating in this study, all patients provided informed, written consent and institutional review board approvals were granted by each site, which was conducted in accordance with the Declaration of Helsinki and Guidelines for Good Pharmacoepidemiology Practices.

### Study participants

Eligible participants consisted of patients with a range of underlying etiologies for spasticity, newly treated with onabotA for spasticity as well as those who had previously received this treatment (onabotA‐naïve patients and onabotA‐nonnaïve patients), patients who were 18 years or older and had received onabotA treatment for spasticity during routine clinical practice at participating sites. Patients who had been treated with botulinum toxins other than onabotA were excluded from the study as were patients who participated in other spasticity clinical trials. Patients with baseline pain (NPRS>0) treated with onabotA for UL, LL, or both UL and LL spasticity at least once during the 2‐year study were included in this post hoc subanalysis. Subgroup analyses included stratification by prior onabotA treatment for spasticity (onabotA‐naïve and onabotA‐nonnaïve patients) and by age categories based on mean and median age of patients (<55 years and ≥55 years patients).

### Control for bias

Because the ASPIRE study was designed to allow generalizability to clinical practice, broad eligibility criteria were implemented in its design to minimize selection bias and evaluate the use of onabotA for spasticity across multiple etiologies and geographic regions, including patients who were both new and experienced with botulinum toxins for spasticity treatment and were from different age groups.

### Outcomes and data

Clinical characteristics and demographics in patients receiving at least one onabotA treatment for spasticity were collected at baseline; onabotA use was collected at each treatment session (Tx) throughout the study. Outcomes assessed were pain measured by NPRS and DAS pain subscale for the UL^46^ and LL,^47^ and patient and physician satisfaction with onabotA treatment for spasticity (Supplemental Figure S1).

NPRS is used to assess pain intensity by rating the level of pain experienced by patients in the past 24 hours using an 11‐point rating scale (range: 0 to 10), where “0” represents no pain and “10” represents the worst pain imaginable.^48^ The baseline NPRS was evaluated at office visit 1 prior to onabotA treatment and reflects the initial measurements available for the patients treated at any subsequent treatments. DAS is an outcome assessed by the physician to measure functional impairment in patients with spasticity. In this study, pain subscale on DAS was included. The DAS uses a 4‐point rating scale, with 0 as no disability; 1, mild disability; 2, moderate disability; 3, severe disability. The baseline DAS was evaluated at office visit 1 prior to first onabotA treatment; Tx2–8 reflect evaluation of the prior treatment. Patient satisfaction responses were collected 5 ± 1 weeks after each treatment via web or phone; a follow‐up interview was conducted 12 weeks after the final treatment. Clinician satisfaction data for each treatment (Tx1–7) were collected at the start of each treatment.

Safety was evaluated throughout the 2‐year study period. Patient‐reported adverse events (AEs) associated with the administration of onabotA treatment for spasticity were categorized and summarized using the Medical Dictionary for Regulatory Activities version 20.0 by system organ class and preferred term. A panel of safety clinicians reviewed and determined any potential relationship between the AEs and onabotA treatment.

### Statistical analysis

Analyses were primarily explorative and descriptive in nature and did not test any prespecified hypotheses with missing data not being imputed. Descriptive statistics were used for presenting demographics. A mixed effects regression model was used to assess NPRS and DAS mean changes for the repeated measurements for each patient over up to eight Tx; *p* values and 95% confidence intervals (CI) were adjusted for multiple comparisons using a multivariate t distribution. Sample sizes at each Tx were extracted from the observed values. For responder end points (≥30%, ≥50%, and ≥70% clinically meaningful reduction in mean NPRS score), logistic regression was also used to determine influential baseline covariates. All analyses were conducted using R version 4.3.0 or greater and the emmeans (estimated marginal means) package version 1.10.0 or greater.

## RESULTS

### Patient disposition, demographics, and clinical characteristics

**FIGURE 1:**
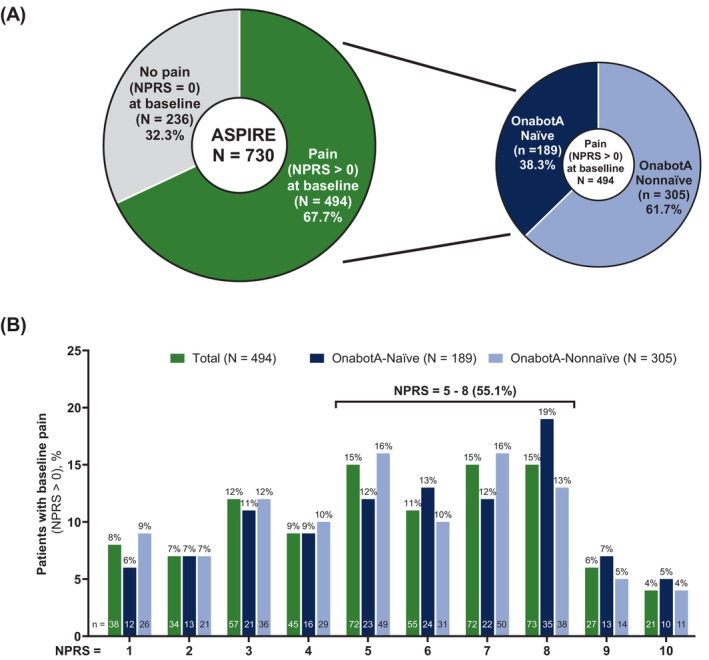
Distribution of patients with spasticity with pain on NPRS >0 (*N* = 494) at baseline in ASPIRE study. NPRS is a validated, patient‐reported measure that rates the level of pain experienced in the last 24 hours from 0 (no pain) to 10 (highest pain imaginable). The baseline NPRS was evaluated at office visit 1. Patients with pain at baseline are presented by (A) overall (green) and by prior onabotA treatment status (naïve – dark blue; nonnaïve – light blue); (B) tabulated NPRS scores (1 to 10) for patients with spasticity experiencing pain for the overall (green), onabotA‐naïve (dark blue), and onabotA‐nonnaïve (light blue). The proportion of overall patients (green bars) reporting NPRS scores between 5 and 8 is indicated above the line. ASPIRE, Adult Spasticity International Registry; N, number of patients in the overall, onabotA‐naïve, and onabotA‐nonnaïve groups; n, number of patients reporting each tabulated NPRS score; NPRS, Numeric Pain Rating Scale; onabotA, onabotulinumtoxinA.

Of the 730 patients enrolled who received ≥1 onabotA dose for spasticity, 494 (67.7%) patients reported pain (NPRS>0) at baseline (Figure 1A) and were included in this post hoc subanalysis (mean [SD] age of 54.0 [15.4] years; predominantly female [56.9%], and largely White [75.9%]) (Table 1). More than half of patients (305/494; 61.7%) received prior onabotA treatment for spasticity with a mean time from prior onabotA treatment for spasticity of 7.7 months. Patients were evenly distributed by age categories (<55 years [244/494; 49.4%] and ≥55 years [250/494; 50.6%]) (Table 1 and Supplemental Table S2). The mean baseline pain score assessed with NPRS was 5.4 with 55.1% (272/494) of patients reporting their NPRS score between 5 and 8 (Figure 1B). Overall, the most common etiology among patients with pain at baseline was stroke (276/494, 55.9%) (Supplemental Figure S2). Similar demographic and clinical characteristic patterns were observed across both treatment subgroups analyzed, onabotA‐naïve and onabotA‐nonnaïve subgroups and <55 years and ≥55 years subgroups (Table 1 and Supplemental Table S2).

**TABLE 1:** Baseline participant demographics and clinical characteristics.

	Total	OnabotA‐naïve	OnabotA‐nonnaïve
	*N* = 494	*n* = 189	*n* = 305
Characteristic			
Age (years), mean (SD)	54. 0 (15.4)	55.6 (15.2)	53.1 (15.4)
Female, *n* (%)	281 (56.9)	99 (52.4)	182 (59.7)
Race or ethnicity, *n* (%)			
American Indian/Alaska Native	1 (0.2)	1 (0.5)	0
Asian	34 (6.9)	18 (9.5)	16 (5.2)
Black/African/Caribbean	59 (11.9)	28 (14.8)	31 (10.2)
White	375 (75.9)	137 (72.5)	238 (78.0)
Latino/Hispanic	13 (2.6)	2 (1.1)	11 (3.6)
Middle Eastern/Arabic	3 (0.6)	1 (0.5)	2 (0.7)
Other	1 (0.2)	0	1 (0.3)
Data not available	8 (1.6)	2 (91.1)	6 (2.0)
Body mass index (kg/m^2^)			
*N* ^a^	401	160	241
Mean (SD)	26.7 (5.8)	27.0 (6.5)	26.6 (5.3)
Spasticity treated^b^, *n* (%)			
Upper limb only	122 (24.7)	46 (24.3)	76 (24.9)
Lower limb only	172 (34.8)	63 (33.3)	109 (35.7)
Upper and lower limb	200 (40.5)	80 (42.3)	120 (39.3)
NPRS pain score			
Total, mean (SD)	5.4 (2.5)	5.7 (2.5)	5.3 (2.4)
Median (min, max)	6.0 (1.0–10.0)	6.0 (1.0–10.0)	5.0 (1.0–10.0)
DAS pain subscale score			
Upper limb, *N* ^a^	491	189	302
Mean (SD)	1.1 (1.1)	1.3 (1.1)	1.0 (1.0)
Lower limb, *N* ^a^	493	189	304
Mean (SD)	1.2 (1.1)	1.2 (1.0)	1.3 (1.1)

Abbreviations: DAS, Disability Assessment Scale; NPRS, Numerical Pain Rating Scale; onabotA, onabotulinumtoxinA.

^a^ Missing data for some participants. Percentages of patients with available data are shown.

^b^ During the Adult Spasticity International Registry study.

### Spasticity presentations and onabotA treatment use

Overall, during the study, most patients were treated for both UL and LL spasticity (200/494; 40.5%), followed by LL only (172/494; 34.8%), and UL only (122/494; 24.7%) (Table 1); this trend was consistent across both subgroups analyzed (Table 1 and Supplemental Table S2). At baseline, the most common UL spasticity presentations were clenched fist (220/351; 62.8%) and flexed elbow (219/351; 62.4%) and the most common LL spasticity presentation was equinovarus foot (301/420; 71.7%) (Supplemental Table S3). The most common clinical presentations are detailed in Supplemental Table S4.

OnabotA usage data are provided in Table 2 and Supplemental Table S5. Overall, the mean dose of onabotA across 8 Tx was 345 U at Tx1 to 464 U at Tx8 (Table 2). The onabotA‐naïve cohort received similar onabotA doses to those administered to the onabotA‐nonnaïve cohort (314 U to 439 U and 356 U to 492 U, respectively) (Table 2). The mean onabotA dose administered to the <55 years cohort (range: 352 to 448 U) was also similar to that administered to the ≥55 years cohort (range: 338 U to 485 U) (Supplemental Table S6).

**TABLE 2:** Total dose of onabotA across treatment sessions—total, onabotA‐naïve, and onabotA‐nonnaïve participants.

	Tx1	Tx2	Tx3	Tx4	Tx5	Tx6	Tx7	Tx8
	*n* = 494	*n* = 418	*n* = 349	*n* = 294	*n* = 223	*n* = 161	*n* = 104	*n* = 28
Dose (units)—total								
Mean	344.8	350.3	360.4	368.0	390.1	372.9	383.7	463.6
(SD)	(196.3)	(186.1)	(181.9)	(190.1)	(184.1)	(175.4)	(166.5)	(200.6)
Min, max	30, 1100	45, 1125	50, 1100	50, 1200	50, 1200	50, 1225	50, 1200	160, 1200

	Tx1	Tx2	Tx3	Tx4	Tx5	Tx6	Tx7	Tx8
	*n* = 189	*n* = 152	*n* = 121	*n* = 95	*n* = 75	*n* = 55	*n* = 42	*n* = 15
Dose (units)—onabotA‐naïve								
Mean	314.2	339.7	334.4	345.4	373.9	389.5	383.5	439.3
(SD)	(179.7)	(190.2)	(155.6)	(156.8)	(164.9)	(183.1)	(151.3)	(153.0)
Min, max	30, 850	50, 1125	50, 1100	50, 1100	50, 1100	50, 1225	50, 800	180, 710

	Tx1	Tx2	Tx3	Tx4	Tx5	Tx6	Tx7	Tx8
	*n* = 305	*n* = 266	*n* = 228	*n* = 199	*n* = 148	*n* = 106	*n* = 62	*n* = 13
Dose (units)—onabotA‐nonnaïve								
Mean	363.7	356.4	374.1	378.8	398.3	364.3	383.9	491.5
(SD)	(203.9)	(183.8)	(193.3)	(203.6)	(193.1)	(171.5)	(177.3)	(248.4)
Min, Max	40, 1100	45, 1000	60, 1030	50, 1200	100, 1200	62, 1225	100, 1200	160, 1200

Abbreviations: OnabotA, onabotulinumtoxinA; Tx, treatment session.

### OnabotA treatment effectiveness outcome measures

#### Pain intensity assessed by NPRS

**FIGURE 2:**
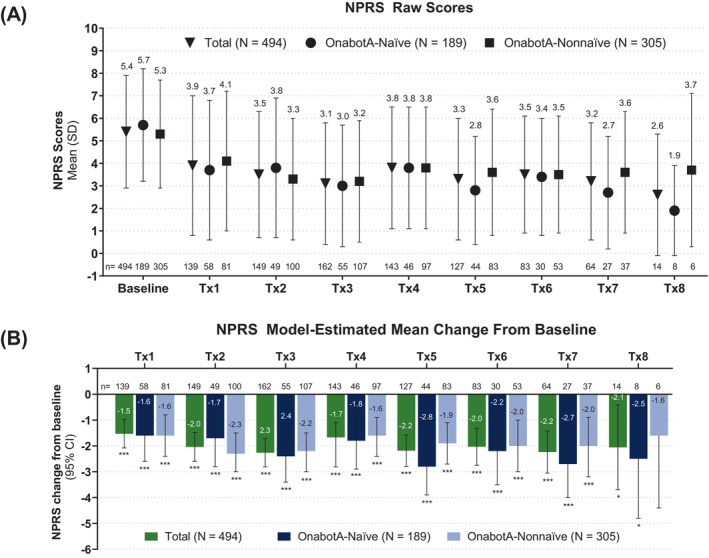
NPRS assessment following onabotA treatment for spasticity across treatment sessions. NPRS is a patient‐reported measure used to assess pain intensity. The baseline NPRS was evaluated at office visit 1 prior to onabotA treatment and reflects the initial measurements for the patients treated at any subsequent treatments. (A) Mean NPRS Scores for total (inverted triangle), onabotA‐naïve (circle), and onabotA‐nonnaïve (square) patients across treatment sessions. Error bars indicate SD. ***p ≤ .0001; *p ≤ .05 (B) Model‐estimated mean change from baseline in NPRS across treatment sessions by overall (green) and by prior onabotA treatment status: onabotA‐naïve patients (dark blue) and onabotA‐nonnaïve patients (light blue). Error bars indicate 95% confidence intervals. CI, confidence interval; n, number of patients; NPRS, Numeric Pain Rating Scale; onabotA, onabotulinumtoxinA; Tx, treatment session.

Following onabotA treatment for spasticity, the mean NPRS scores for pain decreased over time from 5.4 at baseline to 2.6 at Tx8 (Figure 2A). A similar trend in reduction in pain (mean NPRS) from baseline was observed across all Tx regardless of prior onabotA treatment status (Figure 2A) or age group (Supplemental Figure S3A). A decrease from a mean of 5.7 to 1.9 was reported in onabotA‐naïve patients and a decrease from 5.3 to 3.7 was reported in onabotA‐nonnaïve patients. A decrease from 5.7 to 1.7 was reported in the ≥55 years group and a decrease from 5.2 to 3.4 was reported in the <55 years group. The model estimated mean (MEM) NPRS was significantly reduced from baseline (Figure 2B), regardless of prior onabotA exposure (Figure 2B) or age group (Supplemental Figure S3B). MEM NPRS changes between groups (onabotA‐naïve vs. onabotA‐nonnaïve and <55 years vs. ≥55 years cohorts) at each consecutive treatment using the overall cohort mean at baseline revealed no significant difference (Supplemental Table S7).

**FIGURE 3:**
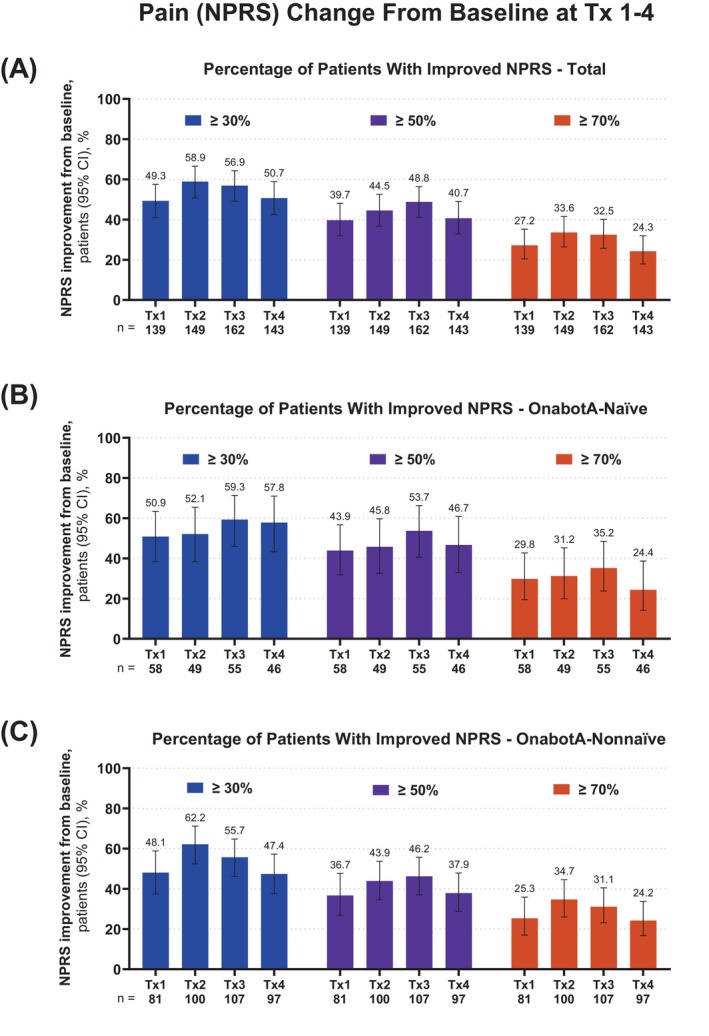
Proportion of patients with spasticity achieving ≥30% (blue), ≥50% (purple), and ≥70% (red) reduction in NPRS from baseline across Tx 1–4. NPRS rates pain intensity experienced by the patient in the past 24 hours from 0 (no pain) to 10 (highest pain imaginable). Data are presented by (A) overall, (B) onabotA‐naïve, and (C) onabotA‐nonnaïve status. The percentage of reduction groups are not mutually exclusive, which is why they add up to >100% for each treatment. The ≥30% reduction group also includes the patients in the ≥50% and ≥70% reduction groups, because 50% and 70% are both ≥30%. The ≥50% reduction group also includes the patients in the ≥70% reduction group, because 70% is ≥50%. Error bars represent 95% confidence intervals. CI, confidence interval; NPRS, Numerical Pain Rating Scale; onabotA, onabotulinumtoxinA; Tx, treatment session.

The proportion of patients with clinically meaningful ≥30%, ≥50%, and ≥70% reduction in pain across Tx1–4 are illustrated for the overall patient population, onabotA‐naïve cohort, and onabotA‐nonnaïve cohort in Figure 3. By Tx4, in the overall patient population, the mean NPRS score was reduced from baseline by ≥30% for 49.3%–58.9% of patients, by ≥50% for 39.7%–48.8% patients, and by ≥70% for 24.3%–33.6% of patients (Figure 3A). Similar clinical meaningful reduction in pain compared to baseline was observed by Tx4 regardless of prior exposure to onabotA (onabotA‐naïve group: the mean NPRS score decreased by ≥30% for 50.9%–59.3% of patients, by ≥50% for 43.9%–53.7% patients, and by ≥70% for 24.4%–35.2% of patients [Figure 3B]; onabotA‐nonnaïve group: the mean NPRS score decreased by ≥30% for 47.4%–62.2% of patients, by ≥50% for 36.7%–46.2% patients, and by ≥70% for 24.2%–34.7% of patients [Figure 3C]).

#### Pain disability measured on DAS

**FIGURE 4:**
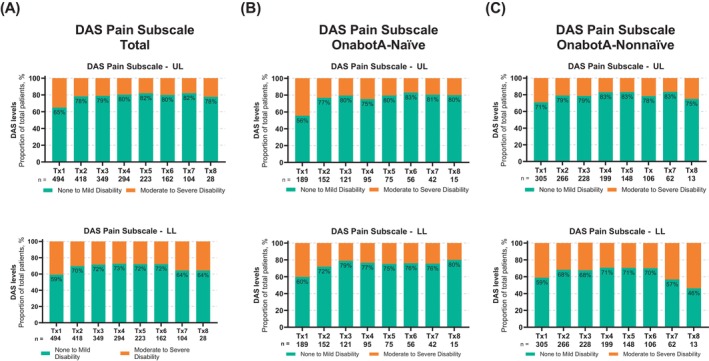
Upper and lower limb disability on the DAS pain subscale across treatment sessions. The DAS uses a 4‐point rating scale, with 0 as no disability; 1, mild disability; 2, moderate disability; 3, severe disability. The baseline DAS was evaluated at office visit 1 (prior to first onabotA treatment); Tx 2–8 reflects evaluation of the prior treatment (ie, Tx 2 reflects the response to Tx 1). (A) Total, (B) onabotA‐naïve, and (C) onabotA‐nonnaïve patients; top panels (UL) and bottom panels (LL). DAS scores of 0–1 indicate none to mild disability (green); DAS scores of 2–3 indicate moderate to severe disability (orange). DAS, Disability Assessment Scale; LL, lower limb; n, number of patients; onabotA, onabotulinumtoxinA; Tx, treatment session; UL, upper limb.

**FIGURE 5:**
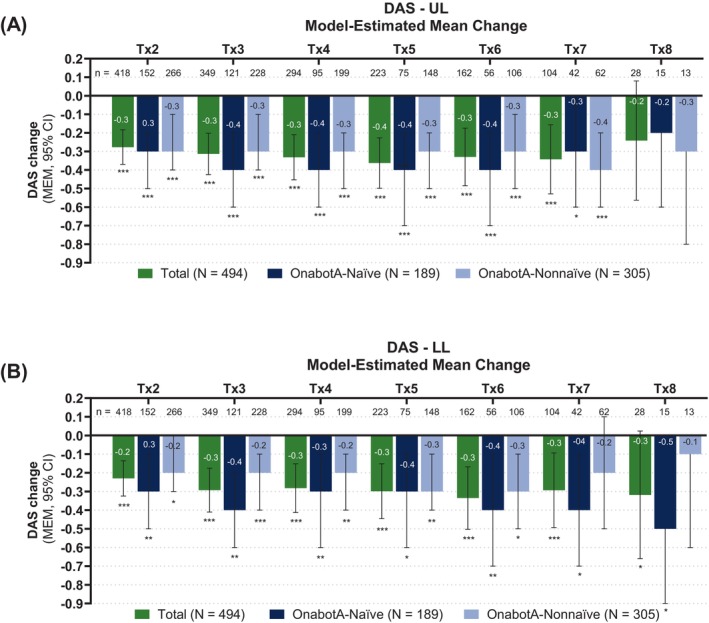
Model‐estimated mean change from baseline for the upper and lower limb DAS pain subscale across treatment sessions. The DAS uses a 4‐point rating scale, with 0 as no disability; 1, mild disability; 2, moderate disability; 3, severe disability The baseline DAS was evaluated by clinicians at office visit 1 (prior to treatment); Tx 2–8 reflects evaluation of the prior treatment. (A) Top graph illustrates mean changes for UL and (B) bottom graph for LL; total (green), onabotA‐naïve (dark blue) and onabotA‐nonnaïve (light blue) patients; error bars indicate 95% confidence intervals; ***p ≤ .0001; **p ≤ .001; *p ≤ .05. CI, confidence interval; DAS, Disability Assessment Scale; LL, lower limb; MEM, model estimated mean; n, number of patients; onabotA, onabotulinumtoxinA; Tx, treatment session; UL, upper limb.

DAS scores on the pain subscale were collected by the treating clinician to assess patients' functional impairment on pain resulting from spasticity (Figure 4). The proportion of patients experiencing no to mild UL and LL disability on the DAS pain subscale increased relative to baseline (≥65% and ≥59%, respectively) across Tx2–8. These levels did not return to baseline throughout the study for UL and LL spasticity (Figure 4A). DAS pain subscale levels for the UL spasticity generally improved relative to baseline across all Tx regardless of prior exposure to onabotA (Figure 4B,C). However, DAS pain subscale levels for the LL spasticity varied for the onabotA‐nonnaïve cohort across treatment sessions (Figure 5C). Improvements on DAS pain subscale compared to baseline were reported for the UL spasticity regardless of age group (Supplemental Figure S4A,B). However, there was a variability in the DAS pain subscale levels reported for the LL spasticity in the ≥55 age group across treatment sessions (Supplemental Figure S4B).

MEM on the DAS pain subscale after onabotA treatment demonstrated significant improvements in UL (Figure 5A) and LL (Figure 5B) pain disability across Tx1–7 compared to baseline in the overall patient population. The MEM DAS for UL and LL spasticity on pain subscale showed significant reduction in pain after onabotA treatment compared to baseline across most Tx regardless of prior exposure to onabotA (Figure 5A,B) or age group (Supplemental Figure S4C). MEM DAS changes between groups (onabotA‐naïve vs. onabotA‐nonnaïve and <55 years vs. ≥55 years cohorts) at each consecutive treatment using the overall cohort mean at baseline revealed no significant differences (Supplemental Table S8).

### Patients' and physicians' treatment satisfaction

**FIGURE 6:**
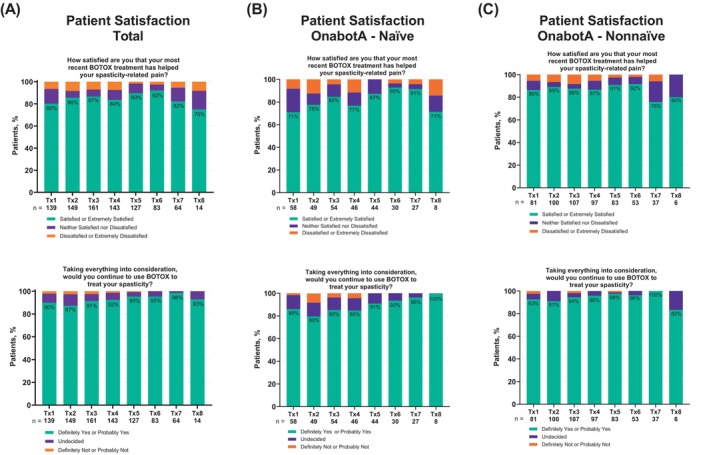
Patient‐reported spasticity related pain reduction and treatment satisfaction with onabotA treatment for spasticity. (A) total, (B) onabotA‐naïve, and (C) onabotA‐nonnaïve patients; N/A responses to top panels (the only question with a N/A option) are not included in vertical bars but are included in the total number of respondents below each treatment. BOTOX, onabotA, onabotulinumtoxinA; n, the number of patients who responded to each question; N/A, not applicable; onabotA, onabotulinumtoxinA; Tx, treatment session.

Overall, across all Tx, most patients (range: 75%–92%) were satisfied or extremely satisfied that their most recent onabotA treatment has helped their spasticity‐related pain (Figure 6A). Patients who responded “not applicable” regarding their satisfaction with onabotA treatment in helping their spasticity‐related pain were not included in the calculation (Figure 6A). Taking everything into consideration, a high proportion of patients (range: 87%–98%) indicated they would continue to use onabotA to treat their spasticity (Figure 6A). Most patients, regardless of prior onabotA treatment for spasticity or age group, were satisfied/extremely satisfied that onabotA treatment helped their spasticity‐related pain (Figure 6B,C, and Supplemental Figure S5A,B) and would continue to use it to treat their spasticity (Figure 6B,C, and Supplemental Figure S5A,B, respectively).

**FIGURE 7:**
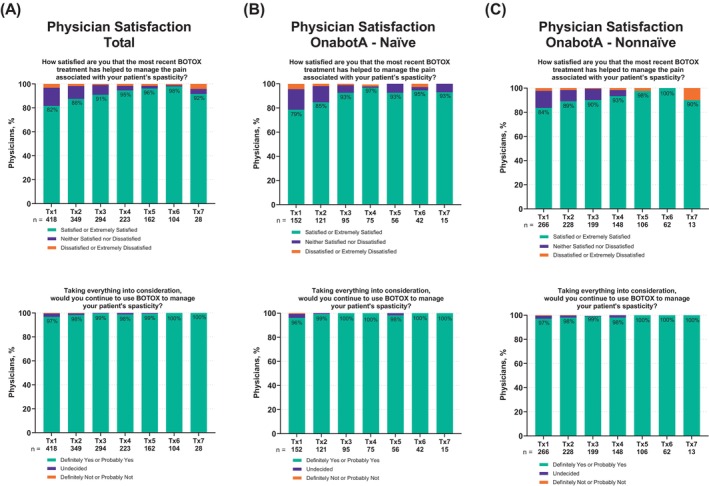
Physician‐reported spasticity related pain reduction and treatment satisfaction with onabotA treatment for spasticity. (A) total, (B) onabotA‐naïve, and (C) onabotA‐nonnaïve patients; N/A responses to top panels (the only question with a N/A option) are not included in vertical bars but are included in the total number of respondents below each treatment. BOTOX, onabotA, onabotulinumtoxinA; n, the number of physicians who responded to each question; N/A, not applicable; onabotA, onabotulinumtoxinA.

Across all Tx, most physicians (range: 82%–98%) reported that they were satisfied/extremely satisfied with how the most recent treatment helped manage their patients' spasticity‐related pain. Physicians who responded “not applicable” were not included in the calculation (Figure 7A). Across Tx, a high proportion of physicians (range: 97%–100%) would continue to use onabotA to manage their patients' spasticity (Figure 7A). Most physicians, regardless of prior onabotA treatment for their patients' spasticity or their patients' age group were satisfied/extremely satisfied that onabotA treatment helped their patients' spasticity‐related pain and would continue to use it to treat their patients' spasticity (Figure 7B,C, and Supplemental Figure S5C,D, respectively).

### Safety profile

A summary of safety data for the overall patient population and across cohort subgroups stratified based on prior onabotA exposure and by age groups is shown in Table 3; all nonserious and serious AEs shown occurring in ≥1% of patients are presented in Supplemental Table S9. Overall, there were 464 nonserious AEs occurring in 172/294 patients (34.8%); 17 nonserious AEs in 15/494 patients (3%) were considered treatment ‐related with muscular weakness reported as the most common treatment‐related AEs (6 events in 6/494 patients, 1.2%). A total of 156 serious AEs occurred in 74/294 patients (15.4%); 3 serious AEs in 2/494 patients (0.4%) were considered treatment related. Dysphagia, muscular weakness, and slow speech treatment‐related serious AEs were reported by one patient each; all three treatment‐related serious AEs occurred in the onabotA‐naïve patients that were ≥ 55 years of age (Table 3).

**TABLE 3:** Summary of participant‐ and event‐based TRAEs and TRSAEs^a^, further stratified by prior onabotA treatment and age groups.

MedDRA terms	Total participants (*N* = 494)		OnabotA‐naive (*N* = 189)		OnabotA‐nonnaive (*N* = 305)		< 55 years (*N* = 244)		≥ 55 years (*N* = 250)	
	Participants *N* (%)^b^	Events *n* ^c^	Participants *N* (%)^b^	Events *n* ^c^	Participants *N* (%)^b^	Events *n* ^c^	Participants *N* (%)^b^	Events *n* ^c^	Participants *N* (%)^b^	Events *n* ^c^
All TRAEs										
Any event	15 (3.0)	17	7 (3.7)	7	8 (2.6)	10	10 (4.1)	10	5 (2.0)	7
Dry mouth	1 (0.2)	1	1 (0.5)	1	0	0	1 (0.4)	1	0	0
Dysphagia	1 (0.2)	1	1 (0.5)	1	0	0	1 (0.4)	1	0	0
Nausea	1 (0.2)	1	0	0	1 (0.3)	1	0	0	1 (0.4)	1
Vomiting	1 (0.2	1	0	0	1 (0.3)	1	0	0	1 (0.4)	1
Asthenia	1 (0.2)	1	1 (0.5)	1	0	0	1 (0.4)	1	0	0
Drug tolerance	1 (0.2)	1	0	0	1 (0.3)	1	1 (0.4)	1	0	0
Gait disturbance	1 (0.2)	1	0	0	1 (0.3)	1	1 (0.4)	1	0	0
Peripheral edema	1 (0.2)	1	0	0	1 (0.3)	1	1 (0.4)	1	0	0
Fall	1 (0.2)	1	0	0	1 (0.3)	1	0	0	1 (0.4)	1
Grip strength decrease	1 (0.2)	1	0	0	1 (0.3)	1	1 (0.4)	1	0	0
Weight increased	1 (0.2)	1	0	0	1 (0.3)	1	0	0	1 (0.4)	1
Muscular weakness	6 (1.2)	6	4 (2.1)	4	2 (0.7)	2	3 (1.2)	3	3 (1/2)	3
All TRSAEs										
Any event	2 (0.4)	3	2 (1.1)	3	0	0	0	0	2 (1.1)	3
Dysphagia	1 (0.2)	1	1 (0.5)	1	0	0	0	0	1 (0.4)	1
Muscular weakness	1 (0.2)	1	1 (0.5)	1	0	0	0	0	1 (0.4)	1
Slow speech	1 (0.2)	1	1 (0.5)	1	0	0	0	0	1 (0.4)	1

Abbreviations: MedDRA, Medical Dictionary for Regulatory Activities; OnabotA, onabotulinumtoxinA; TRAEs, treatment‐related adverse events; TRSAEs, treatment‐related serious adverse events.

^a^ A treatment‐related event is categorized based upon a reasonable possibility of the event being caused by onabotA treatment.

^b^ If a participant had the same event more than once, they are only counted once for the participant counts and percentages.

^c^ Total events include what may be multiple occurrences of the same event for a participant.

## DISCUSSION

There is limited published evidence from randomized controlled trials and observational studies on the role of onabotA in relieving pain in patients with spasticity experiencing pain.^11^, ^12^, ^31^, ^41^, ^42^, ^49^, ^50^, ^51^ Our post hoc subgroup analysis involving patients with spasticity of diverse etiologies, various age groups, and those naïve and nonnaïve to prior exposure to onabotA treatment aligns with previous studies and further supports the significant reduction in pain and functional disability following long‐term onabotA treatment, with high patient‐ and physician‐reported satisfaction. Stroke was the most common underlying etiology in 55.9% of patients and almost two‐thirds of patients were previously treated with onabotA (63.7%). A range of total onabotA doses was used across Tx in the overall patient population (345 U to 464 U), with the maximum dose exceeding the product label of 400 U^28^; this can likely be attributed to the inherent complexities of treating patients in real‐world clinical settings^40^, ^41^, ^42^, ^52^ as the individual dosing at each Tx was decided at the treating physician's discretion tailored to the patient's clinical need.

Outcome measures to determine the effectiveness of onabotA for the treatment of spasticity‐related pain included functional impairment using DAS pain subscale,^46^ pain intensity using NPRS,^48^ and patients' and physicians' satisfaction with treatment. This analysis included patients with spasticity and pain, as indicated by a mean baseline NPRS of 5.4 with more than half of patients (55%) reporting mean NPRS between 5 and 8. Our analysis confirms findings from previous literature, showing that onabotA effectively reduces spasticity‐related pain. In our study, mean NPRS scores decrease from 5.4 at baseline to 2.6 by treatment session eight following onabotA, aligning with previous ASPIRE study results where 275 participants receiving onabotA treatments for both ULs and LLs experienced significant NPRS score reductions from baseline.^31^ A 30% reduction in pain is widely recognized as clinically meaningful in pain management studies, and this threshold is often used to evaluate treatment efficacy.^48^ In our study, a meaningful clinical reduction in pain was achieved in a large proportion of patients across the first four treatments, with 24%–34% of patients experiencing over 70% reduction, 40%–49% experiencing over 50% reduction, and 49%–59% experiencing over 30% reduction of pain. These results, along with a significant reduction in mean pain intensity from baseline across all treatments, further demonstrate a role for onabotA in improving pain outcomes in patients with spasticity.

Patient‐ and physician‐reported satisfaction data were gathered following each onabotA treatment to assess the level of satisfaction with the treatment in reducing pain and willingness to continue onabotA treatment for spasticity. Our data indicates high patient‐ and physician‐reported satisfaction levels with onabotA treatment. In this analysis, ≥75% of patients were satisfied that onabotA treatment helped their pain‐related spasticity, and ≥87% of patients indicated they would continue to use onabotA to treat their spasticity. This trend was observed regardless of prior exposure to onabotA or age groups, suggesting that groups experienced reduction in pain equally. Interestingly, relatively more physicians (≥82%) were satisfied that the onabotA treatment helped their patient's pain‐related spasticity and even a higher proportion (≥97%) indicated they would continue to use onabotA to treat their patient's spasticity across all Tx.

In addition to pain relief, functional impairment as assessed on DAS pain subscale was also improved in our study. DAS scores on pain subscale were significantly reduced compared to baseline in the UL and LL across most Tx, with higher proportions of patients experiencing none to mild UL and LL disability on DAS pain subscale relative to baseline across all Tx. Similar trends were reported in previous ASPIRE studies investigating patients treated with onabotA for UL, LL, and combined limb spasticity,^31^, ^41^, ^42^ where DAS scores showed significant improvement in both pain and functional disability, reinforcing the consistent effectiveness of onabotA in reducing DAS scores across all studies. Subgroup analysis by prior exposure to onabotA and by age group revealed a similar trend in improvements in pain compared to baseline after most onabotA treatments as measured by NPRS and the DAS pain subscale. More important, no significant difference was observed between groups when comparing mean changes in NPRS and DAS pain subscale measures at each treatment. Proposed mechanisms by which onabotA mediates pain include blocking acetylcholine release at the neuromuscular junction causing muscle relaxation and relief from muscle‐overactivity associated pain,^3^, ^39^ and antinociceptive effects by inhibiting the release of other neurotransmitters like glutamate and substance P, involved in pain perception.^38^, ^53^, ^54^ This inhibition leads to an alleviation of peripheral and central sensitization, playing a crucial role in decreasing pain in conditions like CM.^38^ Evidence also suggests that these mechanisms apply to other movement disorders such as CD.^55^ For instance, Tarsy and First (1997) reported that pain levels associated with CD were significantly reduced by onabotA treatment and had not returned to baseline levels at the time of retreatment.^56^ Additional evidence points to a sensory mechanism of action for onabotA in pain relief, which is separate from its muscle‐relaxing effects. For example, the BOTOX Economic Spasticity Trial (BEST) study demonstrated significantly greater pain reduction in patients with poststroke spasticity treated with onabotA compared to placebo. These improvements were sustained over 52 weeks and showed no significant correlation between reductions in muscle tone and pain, indicating a potential decoupling of the antinociceptive and muscle‐relaxing effects of onabotA.^11^

Our findings, along with previous studies, highlight the significant potential of onabotA in managing spasticity‐related pain. The pain relief from onabotA may not be solely dependent on muscle tone reduction. However, it is important to note that our study did not determine the exact type of pain (eg, musculoskeletal pain, neuropathic pain). The absence of an assessment correlating spasticity and pain precludes us from making hypothesis on the exact mechanism underlying pain relief in these patients. Based on the nature of pain experienced by patients, future research should focus on identifying the precise pathways through which onabotA provides pain relief in patients with spasticity, distinguishing between its muscle‐relaxing and/or antinociceptive effects.

## LIMITATIONS

The study has several limitations, including its observational design reflecting real‐world settings with a diverse cohort of patients, limiting the generalizability of the results, which should be interpreted with caution. The study was not placebo controlled or blinded, and the patient‐reported outcomes were self‐evaluations, which could be influenced by patient expectations and lead to bias. Data at later treatments should be interpreted with caution due to a lower sample size for patient‐reported outcomes and challenges in reaching patients. Additionally, functional impairment evaluated on the DAS pain subscale has only been validated as measures for UL spasticity^46^; however, it has also been used in previous studies to evaluate LL spasticity.^31^, ^45^ Furthermore, since the study was conducted before the 2021 updates to the onabotA label in several countries worldwide to include additional muscle injection sites for spasticity treatment, the findings of this study may not completely capture the current potential for pain reduction with the ability to treat more muscles under the new guidelines. It is also important to acknowledge that in the context of the study, spasticity‐associated pain may have limited specificity as patients with spasticity may also experience other types of pain unrelated to their spasticity. Our study is limited by a lack of a classification for pain associated with spasticity, as it did not differentiate between pain localized to spastic muscles and pain experienced in other areas, nor did it distinguish between pain at rest, during exertion, or upon stretching of the affected muscles.

## CONCLUSIONS

The results of this study provide valuable real‐world evidence for pain reduction in patients with spasticity, suggesting that the use of onabotA may play a role in spasticity‐related pain management. Furthermore, the improvement on pain outcomes, assessed by NPRS and DAS, regardless of prior exposure to onabotA or age groups, was paralleled by high patient and physician satisfaction showing that onabotA helped manage spasticity‐related pain and that patients and physicians would continue to use onabotA for the treatment of spasticity. The safety profile for patients with spasticity experiencing pain treated with onabotA was consistent with the known onabotA safety profile with no new safety signals identified in this subgroup of patients from ASPIRE.

## FUNDING INFORMATION

AbbVie funded this study and participated in the study design, research, analysis, data collection, interpretation of data, reviewing, and approval of the publication. All authors had access to relevant data and participated in the drafting, review, and approval of this publication. No honoraria or payments were made for authorship.

## ETHICS STATEMENT

Before participation in this study, patients provided informed, written consent. This study was conducted in accordance with the Declaration of Helsinki and Guidelines for Good Pharmacoepidemiology Practices, as outlined by the International Society for Pharmacoepidemiology. The Independent Ethics Committee or Institutional Review Board (IRB) at each study site approved the study protocol, informed consent forms, and recruitment materials before patient enrollment. The studies were reviewed and approved by the following IRBs: Quorum IRB, University of Missouri IRB, Western IRB, Einstein Healthcare Network IRB, Medical University of South Carolina IRB, University of Maryland IRB, Wheaton Franciscan Healthcare IRB, University of Texas Health Science Center at Houston Committee for the Protection of Human Subjects, Scott & White IRB, Loma Linda University IRB, Penn State Human Subjects Protection Office IRB, University of Pennsylvania IRB, Vanderbilt University IRB, Chang Gung Medical Foundation IRB, Taipei Medical University Joint IRB, Ethik‐Kommission bei der Landesärztekammer Baden‐Württemberg, Ethik‐Kommission der Ärztekammer Westfalen‐Lippe und der Medizinischen Fakultät der Westfälischen Wilhelms‐Universitat, Ethik‐Kommission Albert‐Ludwigs‐Universität Freiburg, Ethik‐Kommission an der Medizinischen Fakütät der Universität Leipzig, Ethik‐Kommission Universitätsklinikum Jena, Medizinische Hochschule Hannover Ethikkommission Vorsitzender, East Midlands‐Derby Research Ethics Committee, Secci on de Ordenaci on Farmacéutica Direcci on General de Ordenaci on y Atenci on Sanitaria, Comité Auton omico de Etica de la Investigaci on de Galicia, CEIC. Parc de Recerca Biomédica, Comite Etico de Invstigaci on Clínica UASP. Programa de Qualitat Assistencial. Subdirecci on Xeral de Farmacia e Produtos Sanitarios. Conselleria de Sanidade Direcci on, Comité Etico de Investigacion Clínica Hospital Mútua de Terrassa, Comité Etico de Investigacion Clínica Institut de Recerca, Comitato Etico Interaziendale Novara, Comitato Etico Interregionale Policlinico di Bari, Comitato Etico Ospedale San Raffaele Ufficio Ricerche Cliniche, Comitato Etico Fondazione Policlinico Universitario “Agostino Gemelli”‐ Università Cattolica del Sacro Cuore. The studies were conducted in accordance with the International Conference for Harmonization guidelines, applicable regulations, and the Declaration of Helsinki.

## DISCLOSURES

AbbVie funded this study and participated in the study design, research, analysis, data collection, interpretation of data, reviewing, and approval of the publication. All authors had access to relevant data and participated in the drafting, review, and approval of publication. No honoraria or payments were made for authorship. Writing assistance was provided by Gina E. Elsen, PhD, of AbbVie. Financial arrangements of the authors with companies whose products may be related to the present manuscript are listed below, as declared by the authors. J. Wissel received honoraria for being on the speaker's bureaus and advisory boards for AbbVie, Ipsen, Medtronic, and Merz; C. List received honoraria for being on the speaker's bureau and advisory board for AbbVie and advisory board for Merz; M. Schwartz is a biostatistical consultant for AbbVie; M. Nelson and T. Musacchio are full‐time employees of AbbVie, Inc. and may hold AbbVie stock; E. Duarte received fees for study participation from Allergan, Dysport, and Merz.

## PRESENTATIONS

Presented in part at the 13th World Congress for Neurorehabilitation, Vancouver, Canada, May 22–25, 2024, and at the 18th World Congress of the International Society of Physical and Rehabilitation Medicine, Sydney, Australia, June 1–6, 2024.

## Supporting information


**Data S1.** Supporting Information.

## Data Availability

AbbVie is committed to responsible data sharing regarding the clinical trials we sponsor. This includes access to anonymized, individual, and trial‐level data (analysis data sets), as well as other information (eg, protocols, clinical study reports, or analysis plans), as long as the trials are not part of an ongoing or planned regulatory submission. This includes requests for clinical trial data for unlicensed products and indications. These clinical trial data can be requested by any qualified researchers who engage in rigorous, independent, scientific research, and will be provided following review and approval of a research proposal, Statistical Analysis Plan (SAP), and execution of a Data Sharing Agreement (DSA). Data requests can be submitted at any time after approval in the United States and Europe and after acceptance of this manuscript for publication. The data will be accessible for 12 months, with possible extensions considered. For more information on the process or to submit a request, visit the following link: https://vivli.org/ourmember/abbvie/ then select “Home”.
