# Supplementary material for: Relief of pain associated with spasticity in adult patients after treatment with onabotulinumtoxinA: Post hoc observational results from the ASPIRE study
Source: PM R. 2025 Sep 30;18(4):410–25. doi: 10.1002/pmrj.70013 (PMC13081772; doi:10.1002/pmrj.70013)
Supplement: Supplementary file 1 — Data S1. Supporting Information. [file PMRJ-18-410-s001.docx]

**Relief of Pain Associated With Spasticity in Adult Patients After Treatment With OnabotulinumtoxinA: Post Hoc Observational Results From the ASPIRE Study**

**SUPPLEMETAL INFORMATION – (5 supplemental figures + 9 supplemental tables)**

**SUPPLEMETAL INFORMATION – FIGURES (5 supplemental figures)**

**Supplemental Figure 1:** Outcomes analyzed in ASPIRE for the subgroup analysis of patients with pain at baseline.

**
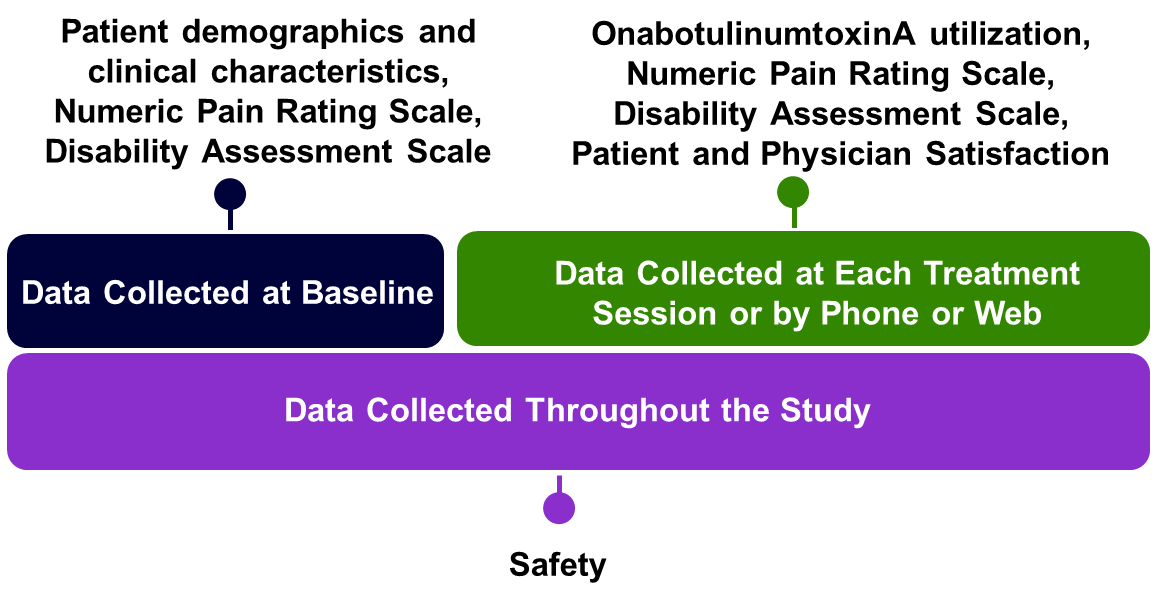
**

**Supplemental Figure 2:** Underlying etiology of spasticity patients at baseline. Etiologies were not mutually exclusive, as more than one etiology could be reported per patient. Stroke includes ischemic, hemorrhagic, and embolic stroke. N, number of patients in the overall (green bar), onabotA-naïve (dark blue), and onabotA-nonnaïve (light blue) patients; n, number of patients in each etiology; onabotA, onabotulinumtoxinA.

**Supplemental Figure 3:** NPRS following onabotA treatment for spasticity across treatment sessions by age groups (<55 years and ≥55 years). NPRS is a patient-reported measure used to assess pain intensity; it rates the level of pain experienced in the last 24 hours using an 11-point rating scale (range: 0 to 10), where "0" represents no pain and "10" represents the worst pain imaginable. The baseline NPRS was evaluated at office visit 1 prior to onabotA treatment and reflects the initial measurements available for the patients treated at any subsequent treatments; response to Tx 2-8 were collected 5 ± 1 weeks post treatment by phone or web after each treatment. (A) Mean NPRS Scores for <55 years group (rhombus) and ≥55 years group (triangle) across treatment sessions. Error bars indicate standard deviation.
****P* ≤ .0001. (B) Model-estimated mean change from baseline in NPRS across treatment sessions presented by <55 years group (blue) and by
≥55 years group (purple). Error bars indicate 95% confidence intervals.

CI, confidence interval; n, number of patients; NPRS, Numeric Pain Rating Scale; onabotA, onabotulinumtoxinA; SD, standard deviation; Tx, treatment sessions.

**Supplemental Figure 4**: Upper and lower limb disability on the DAS pain subscale across Tx age group (<55 years and ≥55 years). The DAS uses a 4-point rating scale, with 0 as no disability; 1, mild disability; 2, moderate disability; 3, severe disability. DAS scores of 0-1 indicate none to mild disability (green); DAS scores of 2-3 indicate moderate to severe disability (orange). The baseline DAS was evaluated at office visit 1 (prior to first onabotA treatment); Tx 2-8 reflects evaluation of the prior treatment (i.e., Tx 2 reflects the response to Tx 1). (A) DAS, <55 years group; top panel, UL and lower panel, UL; (B) DAS, ≥55 years group; top panel, UL and bottom panel, LL; (C) Model-estimated mean change from baseline for the Disability Assessment Scale (DAS) pain subscale; top panels (UL) and bottom panels (LL); <55 years (purple), ≥55 years (blue) patients; error bars indicate 95% confidence intervals; ****P* ≤ .0001; ***P* ≤ .001; **P* ≤ .05.

CI, confidence interval; DAS, Disability Assessment Scale; LL, lower limb; n, number of patients; onabotA, onabotulinumtoxinA; Tx, treatment; UL, upper limb.

**Supplemental Figure 5**: Patient- and physician-reported spasticity related pain reduction and treatment satisfaction with onabotA for spasticity by age group (<55 year ≥55 years). (A, B) patient responses, and (C, D) physician responses. N/A responses to top panels (the only question with a N/A option) are not included in vertical bars but are included in the total number of respondents below each treatment.

BOTOX, onabotA, onabotulinumtoxinA; n, the number of physicians who responded to each question; N/A, not applicable; Tx, treatment.

**SUPPLEMETAL INFORMATION – TABLES ( 9 supplemental tables)**

| **Supplemental Table 1:** Baseline Pain (NPRS) - tabulated across age groups | | |
| --- | --- | --- |
|  | **< 55 years N = 244** | **≥ 55 years  N = 250** |
| **Baseline Pain (NPRS),  Tabulated, n (%)** |  |  |
| 1 | 26 (10.7) | 12 (4.8) |
| 2 | 20 (8.2) | 14 (5.6) |
| 3 | 29 (11.9) | 28 (11.2) |
| 4 | 27 (11.1) | 18 (7.2) |
| 5 | 30 (12.3) | 42 (16.8) |
| 6 | 25 (10.2) | 30 (12.0) |
| 7 | 32 (13.1) | 40 (16.0) |
| 8 | 31 (12.7) | 42 (16.8) |
| 9 | 13 (5.3) | 14 (5.6) |
| 10 | 11 (4.5) | 10 (4.0) |
| NPRS, Numerical Pain Rating Scale | |  |
|  | |  |

| **Supplemental Table 2:** Baseline participant demographics and clinical characteristics stratified by age group | | |
| --- | --- | --- |
|  | **< 55 years** | **≥ 55 years** |
|  | **N = 244** | **N = 250** |
| **Characteristic** |  |  |
| Age, years, mean (SD) | 41.5 (9.9) | 66.3 (8.2) |
| Female, n (%) | 145 (59.4) | 136 (54.4) |
| Race, n (%) |  |  |
| American Indian or Alaska Native | 0 | 1 (0.4) |
| Asian | 21 (8.6) | 13 (5.2) |
| Black/African/Caribbean | 34 (13.9) | 25 (10.0) |
| White | 173 (70.9) | 202 (80.8) |
| Latino/Hispanic | 2 91.1) | 11 93.6) |
| Middle Eastern or Arabic | 2 (0.8) | 1 (0.4) |
| Other | 1 (0.4) | 0 |
| Data not available | 5 (2.0) | 3 (1.2) |
| BMI (kg/m^2^) |  |  |
| N^a^ | 201 | 200 |
| Mean (SD) | 26.6 (6.1) | 26.9 (5.5) |
| **OnabotA naïve, n (%)** | 81 (33.2) | 108 (43.2) |
| **Prior OnabotA treatment for spasticity** | 163 (66.8) | 142 (56.8) |
| **Time from prior onabotA treatment for spasticity, month** |  |  |
| N | 159 | 136 |
| Mean (SD) | 8.3 (18.5) | 7.0 (9.1) |
| **OnabotA within the past 12 weeks, n (%)** | 3 (1.2) | 3 (1.2) |
| **Etiology of spasticity** |  |  |
| Cerebral palsy | 46 918.9) | 7 (2.8) |
| Multiple sclerosis | 47 (19.3) | 39 (15.6) |
| Spinal cord injury | 19 (7.8) | 14 (5.6) |
| Stroke | 100 (41.0) | 176 (70.4) |
| Traumatic brain injury | 19 (7.8) | 7 (2.8) |
| Other etiologies | 25 (10.2) | 20 (8.0) |
| **Spasticity treated^b^, n (%)** |  |  |
| Lower limb only | 99 (40.6) | 73 (29.2) |
| Upper limb only | 44 (18.0) | 78 (31.2) |
| Upper and lower limb | 101 (41.4) | 99 (39.6) |
| **NPRS** |  |  |
| Mean (SD) | 5.2 (2.6) | 5.7 (2.3) |
| Median (Min, Max) | 5.0 (1.0, 10.0) | 6.0 (1.0, 10.0) |
| **DAS Pain Subscale Scores** |  |  |
| Upper limb, N^a^ | 242 | 249 |
| Mean (SD) | 0.9 (1.0) | 1.2 (1.1) |
| Median (Min, Max) | 1.0 (0.0, 3.0) | 1.0 (0.0, 3.0) |
| Lower limb, N^a^ | 243 | 250 |
| Mean (SD) | 1.3 (1.1) | 1.2 (1.1) |
| Median (Min, Max) | 1.0 (0.0, 3.0) | 1.0 (0.0, 3.0) |
| DAS, Disability Assessment Scale; NPRS, Numerical Pain Rating Scale; SD, standard deviation. | | |
| ^a^ Missing data for some participants.  ^b^ During the ASPIRE study | | |

| **Supplemental Table 3:** Clinical presentation at baseline - upper and lower limbs | | | | |
| --- | --- | --- | --- | --- |
|  |  | **Total** | **OnabotA-**  **Naïve** | **OnabotA-Nonnaïve** |
| **Upper limbs clinical presentation, n (%)^a^** | | **N = 351** | **n = 137** | **n = 214** |
|  | Clenched fist | 220 (62.7) | 84 (61.3) | 136 (63.6) |
|  | Flexed elbow | 219 (62.4) | 89 (65.0) | 130 (60.7) |
|  | Flexed wrist | 170 (48.4) | 72 (52.6) | 98 (45.8) |
|  | Adducted/internally rotated shoulder | 125 (35.6) | 53 (38.7) | 72 (33.6) |
|  | Pronated forearm | 108 (30.8) | 42 (30.7) | 66 (30.8) |
|  | Thumb-in-palm | 78 (22.2) | 34 (24.8) | 44 (20.6) |
|  | Intrinsic plus hand | 50 (14.2) | 18 (13.1) | 32 (15.0) |
|  | Other upper limb | 31 (8.8) | 12 (8.8) | 19 (8.9) |
| **Lower limbs clinical presentation, n (%)^a^** | | **N = 420** | **n = 160** | **n = 260** |
|  | Equinovarus foot | 301 (71.7) | 121 (75.6) | 180 (69.2) |
|  | Flexed knee | 122 (29.0) | 47 (29.4) | 75 (28.8) |
|  | Stiff extended knee | 103 (24.5) | 38 (23.8) | 65 (25.0) |
|  | Adducted thigh | 102 (24.3) | 44 (27.5) | 58 (22.3) |
|  | Flexed toe | 96 (22.9) | 36 (22.5) | 60 (23.1) |
|  | Flexed hip | 46 (11.0) | 16 (10.0) | 30 (11.5) |
|  | Striatal/hyperextended/hitchhiker toe | 39 (9.3) | 16 (10.0) | 23 (8.8) |
|  | Other lower limb | 26 (6.2) | 6 (3.8) | 20 (7.7) |
| ^a^ Percentages may be > 100% as patients may have more than one clinical presentation. | | | | |
| OnabotA, onabotulinumtoxinA | |  |  |  |

| **Supplemental Table 4:** Percentage of patients with clinical presentations treated at each treatment session. | | | | | | | | |
| --- | --- | --- | --- | --- | --- | --- | --- | --- |
| **Clinical presentation, n (%)** | **Tx1 n = 494** | **Tx2  n = 418** | **Tx3 n = 349** | **Tx4 n = 294** | **Tx5 n = 223** | **Tx6 n = 161** | **Tx7  n = 104** | **Tx8  n = 28** |
| Adducted/internally rotated shoulder | 93 (18.8) | 87 (20.8) | 65 (18.6) | 58 (19.7) | 52 (23.3) | 36 (22.4) | 22 (21.2) | 8 (28.6) |
| Adducted thigh | 56 (11.3) | 52 (12.4) | 49 (14.0) | 40 (13.6) | 34 (15.2) | 25 (15.5) | 18 (17.3) | 1 (3.6) |
| Clenched fist | 225 (45.5) | 185 (44.3) | 159 (45.6) | 137 (466.6) | 105 (47.1) | 77 (47.8) | 50(48.1) | 15 (53.6) |
| Equinovarus foot | 239 (48.4) | 215 (51.4) | 188 (53.9) | 159 (54.1) | 123 (55.2) | 88 (54.7) | 61 (58.7) | 22 (78.6) |
| Flexed elbow | 209 (42.3) | 166 (39.7) | 139 (39.8) | 1118 (40.1) | 96 (43.0) | 64 (39.8) | 43 (41.3) | 15 (53.6) |
| Flexed hip | 16 (3.2) | 11 (2.6) | 16 (4.6) | 14 (4.8) | 11 (4.9) | 8 (5.0) | 8 (7.7) | 4 (14.3) |
| Flexed knee | 79 (16.0) | 66 (15.8) | 52 (14.9) | 49 (16.7) | 35 (15.7) | 28 (17.4) | 21 (20.2) | 5 (17.9) |
| Flexed toe | 53 (10.7) | 45 (10.8) | 40 (11.5) | 38 (12.9) | 26 (11.7) | 18 (11.2) | 10 (9.6) | 3 (10.7) |
| Flexed wrist | 159 (32.2) | 129 (30.9) | 105 (30.1) | 88 (29.9) | 71 (31.8) | 58 (36.0) | 31 (29.8) | 15 (53.6) |
| Intrinsic plus hand | 41 (8.3) | 39 (9.3) | 36 (10.3) | 34 (11.6) | 28 (12.6) | 17 (10.6) | 12 (11.5) | 5 (17.9) |
| Other lower limb | 29 (5.9) | 22 (5.3) | 19 (5.4) | 20 (6.8) | 14 (6.3) | 13 (8.1) | 5 (4.8) | 3 (10.7) |
| Other upper limb | 41 (8.3) | 38 (9.1) | 42 (12.0) | 33 (11.2) | 32 (14.3) | 28 (17.4) | 21 (20.2) | 5 (17.9) |
| Pronated forearm | 88 (17.8) | 81 (19.4) | 58 (16.6) | 53 (18.0) | 48 (21.5) | 38 (23.6) | 23 (22.1) | 8 (28.6) |
| Stiff extended knee | 51 (10.3) | 50 (12.0) | 40 (11.5) | 36 (12.2) | 26 (11.7) | 17 (10.6) | 11 (10.6) | 6 (21.4) |
| Striatal/hyperextended/hitchhiker toe | 28 (5.7) | 26 (6.2) | 22 (6.3) | 14 (4.8) | 11 (4.9) | 9 (5.6) | 7 (6.7) | 0 (0.0) |
| Thumb-in-palm | 58 (11.7) | 37 (8.9) | 32 (9.2) | 34 (11.6) | 32 (14.3) | 12 (7.5) | 9 (8.7) | 2 (7.1) |
|  | | | | | | | | |

| **Supplemental Table 5:** Total dose of onabotA for lower limb, upper limb, and both lower and upper limb per treatment session. | | | | | | | | |
| --- | --- | --- | --- | --- | --- | --- | --- | --- |
|  | **Tx1** | **Tx2** | **Tx3** | **Tx4** | **Tx5** | **Tx6** | **Tx7** | **Tx8** |
| **Dose upper limb  (total U)** | **n = 122** | **n = 95** | **n = 71** | **n = 56** | **n = 40** | **n = 31** | **n = 16** | **n = 2** |
| Mean  (SD) | 261.0  (149.5) | 279.7 (165.9) | 292.6  (175.6) | 308.0  (198.1) | 332.7  (200.4) | 295.7  (135.0) | 308.8 (118.6) | 170.0  (14.1) |
| Min, max | 40, 750 | 45, 700 | 50, 900 | 60, 1200 | 100, 1190 | 62, 540 | 100, 540 | 160, 180 |
| **Dose lower limb  (total U)** | **n = 172** | **n = 151** | **n = 124** | **n = 104** | **n = 78** | **n = 55** | **n = 38** | **n = 7** |
| Mean  (SD) | 305.4  (174.2) | 323.8 (161.9) | 324.8  (146.0) | 317.6  (134.4) | 339.1 (139.0) | 322.1  (135.9) | 334.2 (136.9) | 447.1  (105.0) |
| Min, Max | 30, 1100 | 50, 1100 | 50, 900 | 50, 662 | 50, 700 | 50, 600 | 50, 570 | 250, 540 |
| **Dose upper and lower limb  (total U)** | **n = 200** | **n = 172** | **n = 154** | **n = 134** | **n = 105** | **n = 75** | **n = 50** | **n = 19** |
| Mean  (SD) | 429.7  (207.1) | 412.7 (197.5) | 420.2  (193.0) | 432.1  (204.3) | 449.8  (190.1) | 442.1  (190.8) | 445.3 (179.4) | 500.5  (213.5) |
| Min, max | 62, 1000 | 50, 1125 | 80, 1100 | 80, 1200 | 200, 1200 | 150, 1125 | 180, 1200 | 225, 1200 |
| OnabotA, onabotulinumtoxinA; SD, standard deviation; Tx, treatment session; U, units. | | | | | |  |  |  |

| **Supplemental Table 6:** Total dose of onabotA across treatment sessions - total, < 55 years, and ≥ 55 years participants. | | | | | | | | |
| --- | --- | --- | --- | --- | --- | --- | --- | --- |
|  | **Tx1** | **Tx2** | **Tx3** | **Tx4** | **Tx5** | **Tx6** | **Tx7** | **Tx8** |
|  | **n = 244** | **n =209** | **n = 173** | **n = 148** | **n = 112** | **n = 89** | **n = 57** | **n = 16** |
| **Dose (U) - < 55 years** | | | | | | | | |
| Mean   (SD) | 351.8 (200.8) | 352.8 (198.6) | 355.3 (197.3) | 372.3 (209.0) | 395.7 (209.8) | 371.1 (194.4) | 381.0  (187.8) | 447.2 (243.3) |
| Min, Max | 40, 1000 | 50, 1125 | 50, 1100 | 50, 1200 | 100, 1200 | 62 1225 | 100. 1200 | 160, 1200 |
|  | **Tx1** | **Tx2** | **Tx3** | **Tx4** | **Tx5** | **Tx6** | **Tx7** | **Tx8** |
|  | **n = 250** | **n = 209** | **n = 176** | **n = 146** | **n = 111** | **n = 72** | **n = 47** | **n = 12** |
| **Dose (U) - ≥ 55 years** | | | | | | | | |
| Mean   (SD) | 337.9 (191.9) | 347.9 (173.1) | 365.3 (165.7) | 363.6 (169.4) | 384.5 (154.5) | 375.1 (149.9) | 387.0 (138.3) | 485.4 (131.0) |
| Min, Max | 30, 1100 | 45, 930 | 50, 830 | 50, 800 | 50, 800 | 50, 650 | 50, 600 | 300, 710 |
| OnabotA, onabotulinumtoxinA; SD, standard deviation; Tx, treatment session; U, units. | | | | | | | | |

| **Supplemental Table 7:** Model-estimated mean NPRS changes between groups at each Tx | | | | |
| --- | --- | --- | --- | --- |
|  | **Change** | **Lower** | **Upper** | ***P* Value** |
|  |  | **95% CI** | **95% CI** |  |
| OnabotA Non-Naïve Tx 1 - Naive Tx 1 | -0.1 | -1.3 | 1.2 | 1.0000 |
| OnabotA Non-Naïve Tx 2 - Naive Tx 2 | -0.5 | -1.8 | 0.8 | 0.9778 |
| OnabotA Non-Naïve Tx 3 - Naive Tx 3 | 0.2 | -1.1 | 1.4 | 1.0000 |
| OnabotA Non-Naïve Tx 4 - Naive Tx 4 | 0.2 | -1.1 | 1.5 | 1.0000 |
| OnabotA Non-Naïve Tx 5 - Naive Tx 5 | 0.9 | -0.4 | 2.3 | 0.5152 |
| OnabotA Non-Naïve Tx 6 - Naive Tx 6 | 0.2 | -1.4 | 1.8 | 1.0000 |
| OnabotA Non-Naïve Tx 7 - Naive Tx 7 | 0.7 | -1.1 | 2.4 | 0.9920 |
| OnabotA Non-Naïve Tx 8 - Naive Tx 8 | 0.9 | -2.8 | 4.5 | 1.0000 |
| ≥ 55 Years Tx 1 - < 55 Years Tx 1 | 0.4 | -1.9 | 1.6 | 0.9997 |
| ≥ 55 Years Tx 2 - < 55 Years Tx 2 | -0.1 | -1.3 | 1.1 | 1.0000 |
| ≥ 55 Years Tx 3 - < 55 Years Tx 3 | 0.6 | -1.1 | 1.8 | 0.8249 |
| ≥ 55 Years Tx 4 - < 55 Years Tx 4 | 0.5 | -1.1 | 1.8 | 0.9604 |
| ≥ 55 Years Tx 5 - < 55 Years Tx 5 | 0.4 | -0.4 | 1.7 | 0.9995 |
| ≥ 55 Years Tx 6 - < 55 Years Tx 6 | 0.4 | -1.4 | 2 | 0.9999 |
| ≥ 55 Years Tx 7 - < 55 Years Tx 7 | 0.1 | -1.1 | 1.9 | 1.0000 |
| ≥ 55 Years Tx 8 - < 55 Years Tx 8 | -0.7 | -2.8 | 2.9 | 1.0000 |
| CI, confidence interval; NPRS, Numerical Pain Rating Scale; onabotA, onabotulinumtoxinA;  Tx, treatment session. | | | | |

| **Supplemental Table 8:** Model-estimated mean DAS upper and lower limbs changes between groups at each Tx using the overall cohort mean at baseline | | | | |
| --- | --- | --- | --- | --- |
|  | **Change** | **Lower** | **Upper** | **P Value** |
|  |  | **95% CI** | **95% CI** |  |
| **OnabotA-Nonnaïve vs Naïve** |  |  |  |  |
| **Upper Limb OnabotA-Nonnaïve vs Naïve** | |  |  |  |
| OnabotA-Nonnaïve Tx 2 vs Naïve Tx 2 | 0.1 | -0.2 | 0.3 | 0.9970 |
| OnabotA-Nonnaïve Tx 3 vs Naïve Tx 3 | 0.2 | -0.1 | 0.4 | 0.5340 |
| OnabotA-Nonnaïve Tx 4 vs Naïve Tx 4 | 0 | -0.2 | 0.3 | 1.0000 |
| OnabotA-Nonnaïve Tx 5 vs Naïve Tx 5 | 0.1 | -0.2 | 0.4 | 0.9920 |
| OnabotA-Nonnaïve Tx 6 vs Naïve Tx 6 | 0.1 | -0.2 | 0.4 | 0.9797 |
| OnabotA-Nonnaïve Tx 7 vs Naïve Tx 7 | -0.1 | -0.4 | 0.3 | 1.0000 |
| OnabotA-Nonnaïve Tx 8 vs Naïve Tx 8 | -0.2 | -0.8 | 0.5 | 0.9991 |
| **Lower Limb OnabotA-Nonnaïve vs Naïve** |  |  |  |  |
| OnabotA-Nonnaïve Tx 2 vs Naïve Tx 2 | 0.1 | -0.2 | 0.3 | 0.9802 |
| OnabotA-Nonnaïve Tx 3 vs Naïve Tx 3 | 0.2 | -0.1 | 0.4 | 0.3687 |
| OnabotA-Nonnaïve Tx 4 vs Naïve Tx 4 | 0.1 | -0.2 | 0.4 | 0.9884 |
| OnabotA-Nonnaïve Tx 5 vs Naïve Tx 5 | 0.1 | -0.3 | 0.4 | 1.0000 |
| OnabotA-Nonnaïve Tx 6 vs Naïve Tx 6 | 0.1 | -0.2 | 0.5 | 0.9672 |
| OnabotA-Nonnaïve Tx 7 vs Naïve Tx 7 | 0.2 | -0.2 | 0.6 | 0.8262 |
| OnabotA-Nonnaïve Tx 8 vs Naïve Tx 8 | 0.4 | -0.3 | 1.1 | 0.7624 |
| **≥ 55 Years vs < 55 Years** |  |  |  |  |
| **Upper Limb ≥ 55 Years vs < 55 Years** |  |  |  |  |
| ≥ 55 Years Tx 2 vs < 55 Years Tx 2 | 0.1 | -1.3 | 1.1 | 1.0000 |
| ≥ 55 Years Tx 3 vs < 55 Years Tx 3 | 0.1 | -1.1 | 1.8 | 0.8249 |
| ≥ 55 Years Tx 4 vs < 55 Years Tx 4 | 0.1 | -1.1 | 1.8 | 0.9604 |
| ≥ 55 Years Tx 5 vs < 55 Years Tx 5 | 0 | -0.4 | 1.7 | 0.9995 |
| ≥ 55 Years Tx 6 vs < 55 Years Tx 6 | 0 | -1.4 | 2 | 0.9999 |
| ≥ 55 Years Tx 7 vs < 55 Years Tx 7 | 0 | -1.1 | 1.9 | 1.0000 |
| ≥ 55 Years Tx 8 vs < 55 Years Tx 8 | 0 | -2.8 | 2.9 | 1.0000 |
| **Lower Limb ≥ 55 Years vs < 55 Years** |  |  |  |  |
| ≥ 55 Years Tx 2 vs < 55 Years Tx 2 | -0.1 | -0.3 | 0.2 | 0.9989 |
| ≥ 55 Years Tx 3 vs < 55 Years Tx 3 | 0.1 | -0.2 | 0.3 | 0.9949 |
| ≥ 55 Years Tx 4 vs < 55 Years Tx 4 | 0.1 | -0.2 | 0.4 | 0.9887 |
| ≥ 55 Years Tx 5 vs < 55 Years Tx 5 | 0 | -0.3 | 0.3 | 1.0000 |
| ≥ 55 Years Tx 6 vs < 55 Years Tx 6 | 0.2 | -0.2 | 0.5 | 0.7615 |
| ≥ 55 Years Tx 7 vs < 55 Years Tx 7 | 0.2 | -0.2 | 0.6 | 0.7399 |
| ≥ 55 Years Tx 8 vs < 55 Years Tx 8 | 0.2 | -0.5 | 0.9 | 0.9997 |
| CI, confidence interval; DAS, Disability Assessment Scale; onabotA, onabotulinumtoxinA;  Tx, treatment session. | | | | |

| **Supplemental Table 9:** All nonserious and serious adverse events | |  |
| --- | --- | --- |
| **MedDRA terms** | **Total Participants  (N = 494)** | |
|  | **Participants^a^ n (%)** | **Events^b^, n** |
| **All non-serious AEs^a^ (in ≥ 1.0% of patients)** | **172 (34.8)** | **464** |
| Fall | 28 (5.7) | 33 |
| Muscular weakness | 17 (3.4) | 17 |
| Back pain | 13 (2.6) | 15 |
| Arthralgia | 9 (1.8) | 10 |
| Peripheral edema | 8 (1.6) | 8 |
| Depression | 8 (1.6) | 8 |
| Musculoskeletal pain | 8 (1.6) | 9 |
| Multiple sclerosis relapse | 7 (1.4) | 7 |
| Gastroesophageal reflux disease | 6 (1.2) | 6 |
| Bronchitis | 6 (1.2) | 7 |
| Pain in extremity | 6 (1.2) | 6 |
| Muscle spasticity | 6 (1.2) | 6 |
| Seasonal allergy | 5 (1.0) | 5 |
| Cellulitis | 5 (1.0) | 5 |
| Viral upper respiratory tract infection | 5 (1.0) | 5 |
| Muscle spasms | 5 (1.0) | 6 |
| Constipation | 5 (1.0) | 6 |
| Dizziness | 5 (1.0) | 5 |
| Hypertension | 5 (1.0) | 1 |
| **All non-serious AEs^a^ (in n > 1 patient)** | **76 (15.4)** | **156** |
| Pneumonia | 6 (1.2) | 6 |
| Urinary tract infection | 6 (1.2) | 8 |
| Myocardial infarction | 3 (0.6) | 3 |
| Abdominal pain | 2 (0.4) | 2 |
| Asthenia | 2 (0.4) | 2 |
| Non-cardiac chest pain | 2 (0.4) | 2 |
| Cellulitis | 3 (0.6) | 5 |
| Septic shock | 2 (0.4) | 2 |
| Fall | 3 (0.6) | 3 |
| Fibula fracture | 2 (0.4) | 2 |
| Foot fracture | 2 (0.4) | 2 |
| Hip fracture | 2 (0.4) | 2 |
| Cerebrovascular accident | 3 (0.6) | 3 |
| Encephalopathy | 2 (0.4) | 2 |
| Multiple sclerosis relapse | 4 (0.8) | 4 |
| Muscle spasticity | 2 (0.4) | 2 |
| Seizure | 3 (0.6) | 3 |
| Acute kidney injury | 3 (0.6) | 3 |
| Dyspnea | 2 (0.4) | 3 |
| Respiratory failure | 2 (0.4) | 2 |
| Decubitus ulcer | 2 (0.4) | 2 |
| ^a^ If a participant had the same event more than once, they are only counted once for the  participant counts and percentages. | | |
| ^b^ Total events include what may be multiple occurrences of the same event for a participant. | | |
| AE, adverse event; MedDRA, Medical Dictionary for Regulatory Activities Terminology. | | |
